# RETRACTION: Predictive Value of Preoperative Neutrophil to Lymphocyte Ratio and Platelet to Lymphocyte Ratio Combined With Operating Room Factors for Surgical Site Infection After Laparoscopic Radical Nephrectomy in Renal Cell Carcinoma Patients

**DOI:** 10.1111/iwj.70600

**Published:** 2025-04-21

## Abstract

**RETRACTION:** ShenJ. , XieX. , MengY. , and MuY. , “Predictive Value of Preoperative Neutrophil to Lymphocyte Ratio and Platelet to Lymphocyte Ratio Combined With Operating Room Factors for Surgical Site Infection After Laparoscopic Radical Nephrectomy in Renal Cell Carcinoma Patients,” International Wound Journal 21, no. 1 (2024): e14400, 10.1111/iwj.14400.37718121 PMC10788578

The above article, published online on 17 September 2023, in Wiley Online Library (http://onlinelibrary.wiley.com/), has been retracted by agreement between the journal Editor in Chief, Professor Keith Harding; and John Wiley & Sons Ltd. Following an investigation by the publisher, all parties have concluded that this article was accepted solely on the basis of a compromised peer review process. The editors have therefore decided to retract the article. The authors did not respond to our notice regarding the retraction.

**RETRACTION:** J. Shen , X. Xie , Y. Meng , and Y. Mu , “Predictive Value of Preoperative Neutrophil to Lymphocyte Ratio and Platelet to Lymphocyte Ratio Combined With Operating Room Factors for Surgical Site Infection After Laparoscopic Radical Nephrectomy in Renal Cell Carcinoma Patients,” International Wound Journal 21, no. 1 (2024): e14400, 10.1111/iwj.14400.37718121 PMC10788578

The above article, published online on 17 September 2023, in Wiley Online Library (http://onlinelibrary.wiley.com/), has been retracted by agreement between the journal Editor in Chief, Professor Keith Harding; and John Wiley & Sons Ltd. Following an investigation by the publisher, all parties have concluded that this article was accepted solely on the basis of a compromised peer review process. The editors have therefore decided to retract the article. The authors did not respond to our notice regarding the retraction.

